# Altered Immunohistochemical Expression of Mast Cell Tryptase and Chymase in the Pathogenesis of Oral Submucous Fibrosis and Malignant Transformation of the Overlying Epithelium

**DOI:** 10.1371/journal.pone.0098719

**Published:** 2014-05-29

**Authors:** Archana Yadav, Rajiv S. Desai, Bansari A. Bhuta, Jatinder S. Singh, Reema Mehta, Akash P. Nehete

**Affiliations:** Department of Oral Pathology, Nair Hospital Dental College, Mumbai, India; Barts & The London School of Medicine and Dentistry, Queen Mary University of London, United Kingdom

## Abstract

Mast cells (MCs) expressing serine proteases; tryptase and chymase, are associated with fibrosis in various diseases. However, little is known about their involvement in oral submucous fibrosis (OSF). Our goal was to evaluate the role of MC tryptase and chymase in the pathogenesis of OSF and its malignant transformation. Immunohistochemical expression of MC tryptase and chymase was evaluated in 20 cases of OSF, 10 cases of oral squamous cell carcinoma (OSCC) and 10 cases of healthy controls. Subepithelial zone of Stage 1 and 2 while deep zone of Stage 3 and 4 OSF demonstrated increased tryptase positive MCs. OSCC revealed a proportionate increase in tryptase and chymase positive MCs irrespective of areas of distribution. An altered balance in the subepithelial and deep distribution of tryptase and chymase positive MCs play an important role in the pathogenesis of OSF and its malignant transformation.

## Introduction

Oral submucous fibrosis (OSF) is a chronic progressive, areca nut chewing habit related, precancerous condition of the oral mucosa predominantly affecting Indians and South Asians. It is clinically characterized by burning sensation of the oral mucosa accompanied by pallor and progressive, irreversible fibrosis leading to difficulty in opening mouth, speech and swallowing [1]. Characteristic histopathologic features of this disease include epithelial atrophy with loss of rete ridges, reduced vascularity, chronic inflammatory infiltrate and hyalinization of the submucosal tissue. The pathogenic mechanism in OSF begins primarily in the connective tissue and epithelial response secondarily. The characteristic fibro-elastic changes observed in the connective tissue are almost entirely due to abnormal accumulation of collagen. OSF is thus regarded as a collagen related disorder induced by betel nut/betel quid chewing habit often resulting in an overall increased production of collagen with decreased collagen degradation [2].

In normal wound healing fibroblasts are transiently activated into myofibroblast, a particular type of fibroblast to proliferate and deposit the collagen [3]. OSF is similar to a wound where continuous signals for tissue repair are emitted. These continuous signals can lead to abnormal production of cytokines and growth factors, resulting in chronic, sustained long term myofibroblast activation leading to fibrosis. Research has shown that fibrosis in OSF is a continuous, scarring process in which the myofibroblast plays an essential role [4]. However, the dynamics of extra cellular matrix remodeling with OSF is largely unknown, since the origin of fibroblast activation in OSF is debated. Chronic inflammatory conditions can evolve a fibrotic phenotype often associated with an increase in the number of MCs, which are the local residents of connective tissue. Several lines of evidence suggest that MC when activated, secrete a large number of fibrogenic factors and have been implicated in the development of various fibrotic conditions affecting the lungs [5], [6], [7], liver [8], [9], skin [10], [11], and kidney [12]. Despite the potential of MCs to mediate fibrosis, limited attention has been given to the role of MCs in OSF. There have been a few studies assessing MC density in OSF using Toluidine blue stain [13], [14], [15], [16], [17], [18] and C-kit [19], which have yielded controversial results. MCs produce and store various profibrotic cytokines including transforming growth factor-β (TGFβ) [20], fibroblast growth factor (FGF), platelet derived growth factor (PDGF), interleukin 1 and 6 (IL-1 & IL-6), and tumor necrosis factor–α (TNF-α) [21]. The significance of these profibrotic cytokines in OSF has been studied extensively in the literature [22], [23], [24], [25].

Human MCs also contain two types of serine protease, tryptase and chymase. Tryptase is a trypsin-like enzyme, and chymase is a chymotrypsin-like enzyme. According to their protease content, human MCs are divided into two phenotypes: Mast cell secreting both tryptase and chymase are termed MC_TC_, while mast cell secreting only tryptase are termed MC_T_. Both MC_T_ and MC_TC_ phenotypes are present in all human tissues. However, the ratio is different in each anatomical site: while MC_TC_ predominates in the skin, heart, synovial and small intestinal submucosa, MC_T_ predominates in the lungs and the small intestinal mucosa [26]. MC tryptase and chymase, the most abundant profibrotic cytokines of human MC have been studied in various fibrotic disorders [5], [8], [10], [12], however, their role in OSF has not been reported so far in the literature.

It is already known that neoangiogenesis is required for the growth and spread of tumor [27], [28]. Increased angiogenesis has been associated with neoplastic progression, metastasis and outcome in several studies in numbers of malignancies [29], [30], [31], [32]. Current literature suggests the probable role of MCs in tumor angiogenesis, thereby favoring the tumor progression [33], [34]. However, the contribution of proangogenic cytokines namely MC tryptase and chymase during the malignant transformation of OSF is not clear yet.

The present study was undertaken to quantify and characterize the distribution of MC subpopulation in OSF, thereby evaluating the potential role of MC tryptase and chymase in the pathogenesis of OSF & its malignant transformation.

## Materials and Methods

### Human Tissue Specimen Collection

**Figure 1:**
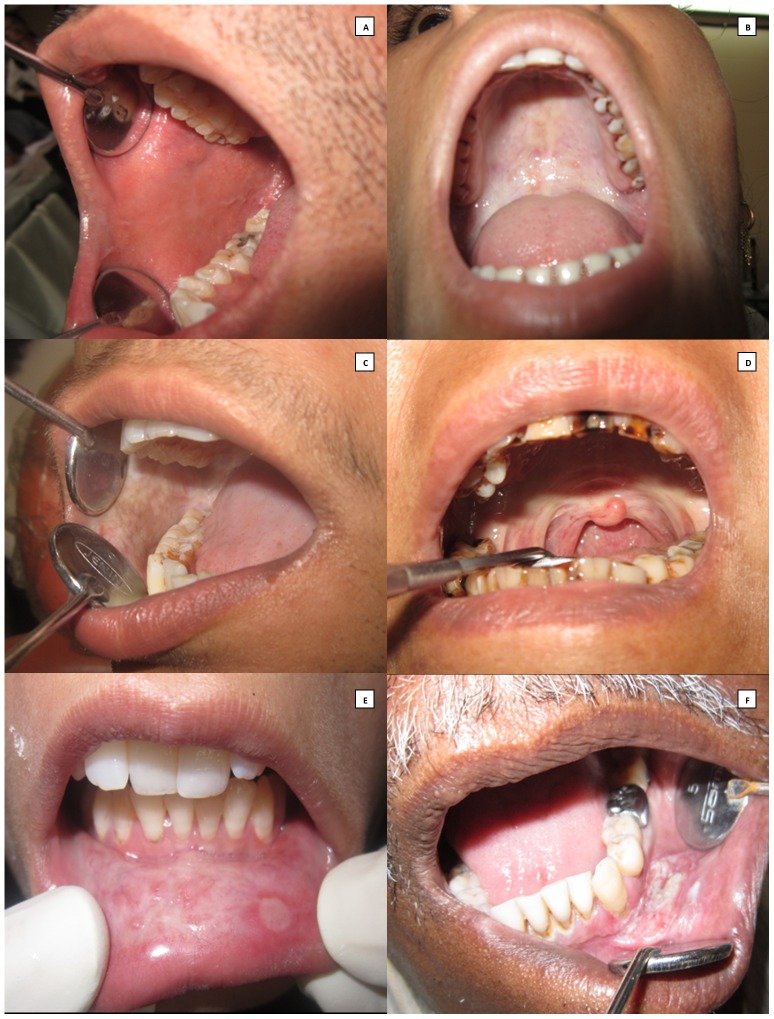
Clinical picture. A, Healthy control. B, Stage 1 OSF: Blanched palatal mucosa with vesicle. **C**, Stage 2 OSF: Blanched buccal mucosa. **D**, Stage 3 OSF: Hockey stick uvula. **E**, Stage 4 OSF: Blanched labial mucosa with vesicle. **F**, OSCC: Carcinoma of the left labial mucosa.

The study protocol was approved by the Institutional Review Board and the Local Ethics Committee of Nair Hospital Dental College and was in compliance with ethical standards of the Declaration of Helsinki. Written informed consents were obtained from all the study participants. Twenty (n = 20) previously untreated cases of OSF and ten (n = 10) cases of OSCC with no history of OSF, diagnosed on clinical grounds and confirmed histologically were randomly selected to form the study groups. Ten (n = 10) age and sex matched healthy volunteers without habits were included in the control group. Patients with mouth opening of more than 40 mm were considered as normal for healthy controls The staging of the disease was performed based upon the degree of mouth opening, Stage 1: mouth opening between 35 and 40 mm; Stage 2: mouth opening between 30 and 34 mm; Stage 3: mouth opening between 20 and 29 mm and Stage 4: mouth opening of less than 20 mm (Figure. 1).

**Figure 2:**
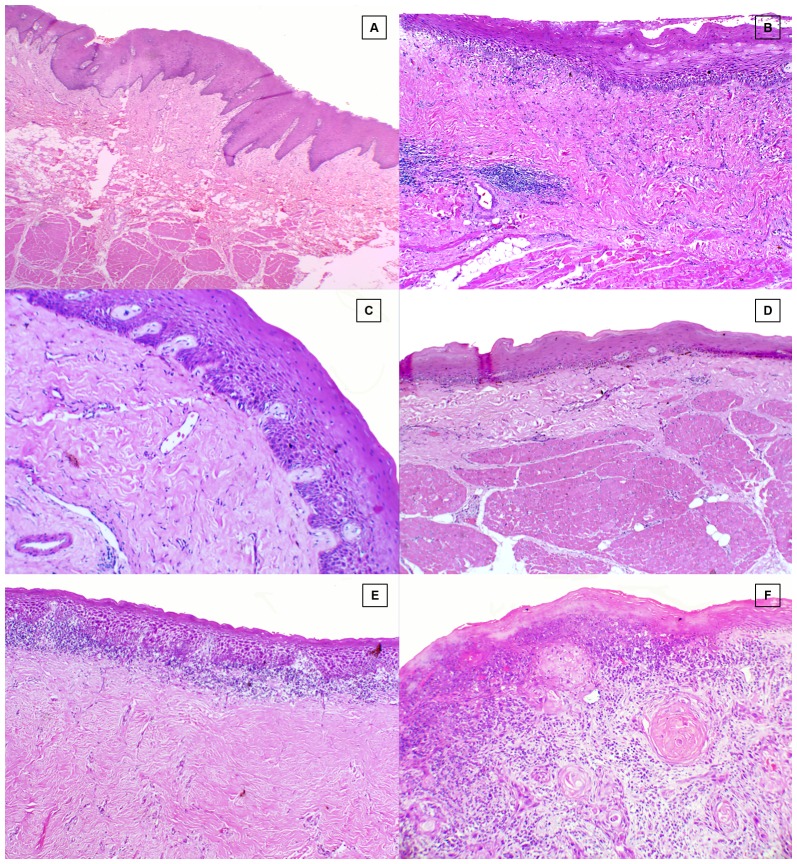
Hematoxylin & eosin stained section. **A**, Healthy control. **B**, Stage1 OSF. **C**, Stage 2 OSF. **D**, Stage 3 OSF. **E**. Stage 4 OSF. F, OSCC.

Punch biopsies (5 mm) were performed on OSF patients and control subjects from identical oral site (right buccal mucosa), whereas for OSCC study group the formalin fixed paraffin blocks were retrieved from the department archives and stained with hematoxylin-eosin for histological examination (Figure. 2)

### Immunohistochemistry

Four-µm-thick formalin fixed paraffin embedded tissue sections were deparafinized in xylene and rehydrated through decreasing graded ethanol solution. Endogenous peroxidase activity was suppressed by incubation for 10 min with 3% hydrogen peroxidase (Dako). The primary monoclonal antibodies used for MC were anti-MC tryptase (Dako) and anti-MC chymase (Abcam). Staining was done at room temperature on automatic staining work station (Dako Autostainer Plus) using the Dako Envision Flex Plus Visualization System (Dako). Counterstaining with hematoxylin was the final step. Normal skin sections were used as positive controls and a negative control was performed in all cases by omitting the primary antibody.

### Immunohistochemical evaluation of MC tryptase and chymase

Tryptase and chymase positive MCs were counted separately in serial sections of healthy controls, OSF and OSCC using Carl Zeiss, Axiolab microscope. Cytoplasm showing brown colour was considered positive immunoreactivity. MCs were counted in two areas of immunostained sections, namely subepithelial and deep. MC density was assessed in areas showing the highest concentration of tryptase and chymase positive MC (hot spots) as determine by an initial scan at 100X magnification (10X objective and 10X Ocular lens). MCs were then counted from 5 different fields at 400X magnification using an ocular grid of 12.5×12.5 mm size divided into 100 blocks.

### Statistical analysis

Statistical analysis was performed using the SPSS software for Windows S version 16.0 (SPSS Inc., Chicago, IL, USA). The statistical analysis was carried out using Kruskal-Wallis test to compare mean values (cases, within cases and controls) and Mann Whitney U test for individual mean values. For all tests, p-values <0.05 were considered to be statistically significant.

## Results

The present study consisted of 20 OSF, 10 OSCC and 10 healthy controls. There were 5 cases in each stage of OSF (Stage 1 to Stage 4). The patients with OSF were in age group of 18 to 75 years, with the mean age being 33.9 years with a marked male predominance (male to female ratio of 13∶7) (Table 1) There are two populations of mast cells, those containing only tryptase (MC_T_) and those containing both tryptase and chymase (MC_TC_), only chymase containing MCs are extremely rare, therefore all MCs contain tryptase [21], [35]. Tryptase positive MCs represented total MCs, while the number of chymase positive MCs can be considered equal to number of MC_TC_. To determine MC subpopulations (MC_T_, MC_TC_) in healthy controls, OSF and OSCC, the formula: Total MCs = MC_T_+MC_TC_ was used as previously described [36]. In all tissues examined, the predominant MC population was positive for both tryptase and chymase. (Table 2).

**Table 1:** Relation of mast cell tryptase and chymase to clinical parameters among the healthy controls, OSF and OSCC.

Sr No	Age	Sex	MO (mm)	Study groups	Mean SeT	Mean DT	MeanSeC	Mean DC
1	38	M	48	Healthy Control	9.8	3	7.6	5
2	30	F	45	Healthy Control	4.8	4.8	6.6	5
3	30	M	44	Healthy Control	13	6.6	16.4	7
4	37	F	44	Healthy Control	9.8	7.4	7.2	4.8
5	57	M	42	Healthy Control	13.2	8.2	9.6	7.4
6	26	F	48	Healthy Control	9	7.6	6.4	7
7	27	F	48	Healthy Control	15.6	8.6	9.4	5.4
8	45	M	48	Healthy Control	10.4	6.8	9	6.6
9	41	F	44	Healthy Control	21.4	5.4	12.6	6.2
10	57	M	49	Healthy Control	10	6.8	13	7.2
11	26	M	39	Stage 1 OSF	14.6	5	6.4	3.4
12	55	M	36	Stage 1 OSF	14.4	4.8	5	3.8
13	32	F	32	Stage 1 OSF	13	2.6	6.8	2.6
14	47	M	39	Stage1 OSF	18.8	4.4	13	2.8
15	18	M	39	Stage 1 OSF	9.2	4.4	5.8	4.2
16	25	M	34	Stage 2 OSF	10.6	5	8.4	6
17	32	M	32	Stage 2 OSF	9.8	4.4	4	2.4
18	35	F	33	Stage 2 OSF	4.8	3	2.4	2.8
19	40	M	34	Stage 2 OSF	5.2	4	4	2.6
20	32	M	33	Stage 2 OSF	4.8	4.2	1.6	1.2
21	30	F	27	Stage 3 OSF	5	6.6	3.6	4.8
22	21	F	21	Stage 3 OSF	3.2	6.4	3	4.6
23	30	M	28	Stage 3 OSF	5.6	9.6	4.2	4.6
24	24	M	27	Stage 3 OSF	4.8	8.2	3	5.4
25	24	M	23	Stage 3 OSF	7.4	6.8	9.6	5.6
26	46	F	7	Stage 4 OSF	4	13.4	1.4	1.4
27	56	M	Nil	Stage 4 OSF	4.8	9.2	3.6	5.2
28	38	F	4	Stage 4 OSF	9.2	8.4	6	3
29	25	F	9	Stage 4ODF	6	5.6	4	3.6
30	42	M	Nil	Stage 4 OSF	6.8	7.6	3.8	4.6
31	65	M	47	OSCC	15	16.4	12.8	12.6
32	57	M	45	OSCC	8.8	9.6	17.2	6.6
33	86	M	44	OSCC	6.6	6.6	4.8	6.2
34	65	M	44	OSCC	12.4	11	8	12.6
35	75	M	42	OSCC	13.6	14.2	15.4	10.6
36	55	M	47	OSCC	10.6	11	13.6	16.8
37	50	M	45	OSCC	12.2	13.8	13	7.8
38	47	M	48	OSCC	18.2	19.2	12.6	5.2
39	62	M	44	OSCC	14.4	16.4	16.6	2.6
40	59	M	49	OSCC	34.8	19	22	20.8

M, male; F, female; SeT, subepithelial tryptase; DT, deep tryptase; SeC, subepithelial chymase; DC, deep chymase; OSF, oral submucous fibrosis; OSCC, oral squamous cell carcinoma.

**Table 2:** Subepithelial and deep distribution of mean MC_T_ and MC_TC_ counts in the healthy controls, OSF and OSCC.

	TMC	MC_TC_	MC_T_
Subepithelial			
Healthy controls	11.68	9.78	1.9
OSF	8.10	4.98	3.12
OSCC	15.66	13.60	2.06
Deep			
Healthy controls	6.52	6.16	0.36
OSF	5.68	3.68	2.00
OSCC	13.72	12.18	1.54

TMC, total mast cells; MC_TC_, tryptase and chymase positive mast cells; MC_T_, tryptase positive mast cells.

### Subepithelial and deep distribution of tryptase positive MCs and chymase positive MCs between healthy controls, OSF and OSCC group

**Figure 3:**
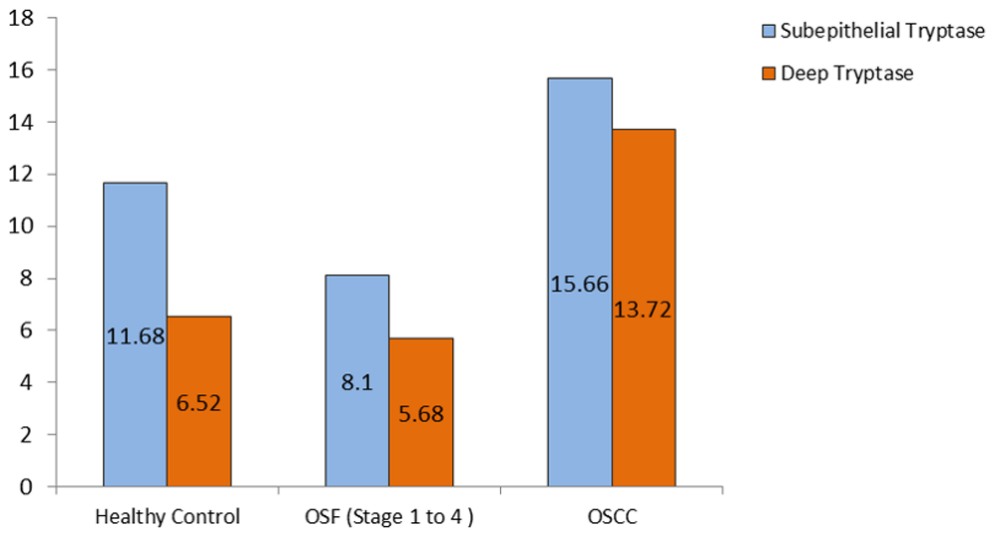
Mean value comparison of subepithelial and deep tryptase positive mast cells in healthy controls, OSF, and OSCC.

We found a statistically significant increase in the number of subepithelial as well as deeper distribution of tryptase positive MCs in OSCC group when compared to OSF group (p<0.05). Subepithelial and deeper distribution of tryptase positive MCs in OSF group demonstrated no statistical significance when compared to healthy controls. From our results, it can be comprehended that OSCC group has significantly more number of tryptase positive MCs as compared to OSF group irrespective of the area of distribution (Figure. 3).

**Figure 4:**
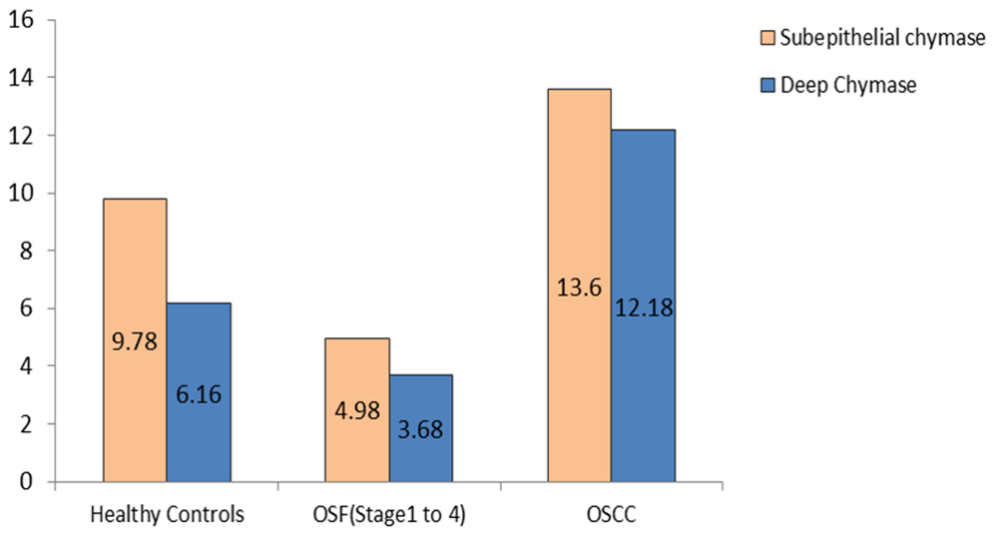
Mean value comparison of subepithelial and deep chymase positive mast cells in healthy controls, OSF, and OSCC.

We observed a statistically significant increase in the number of subepithelial distribution of chymase positive MCs in OSCC group when compared to healthy controls and OSF group (p<0.05). Conversely, a statistically significant decrease in the number of subepithelial distribution of chymase positive MCs in OSF group was noted when compared to healthy controls (p<0.05). Hence, it can be concluded that OSF has the least number of chymase positive MCs subepithelially. We recorded a statistically significant decrease in the number of deep distribution of chymase positive MCs in the OSF group when compared to healthy controls and OSCC group (p<0.05) (Figure. 4).

### Subepithelial distribution of tryptase positive MCs in healthy controls, different stages of OSF and OSCC group (Figure. 5.)

**Figure 5:**
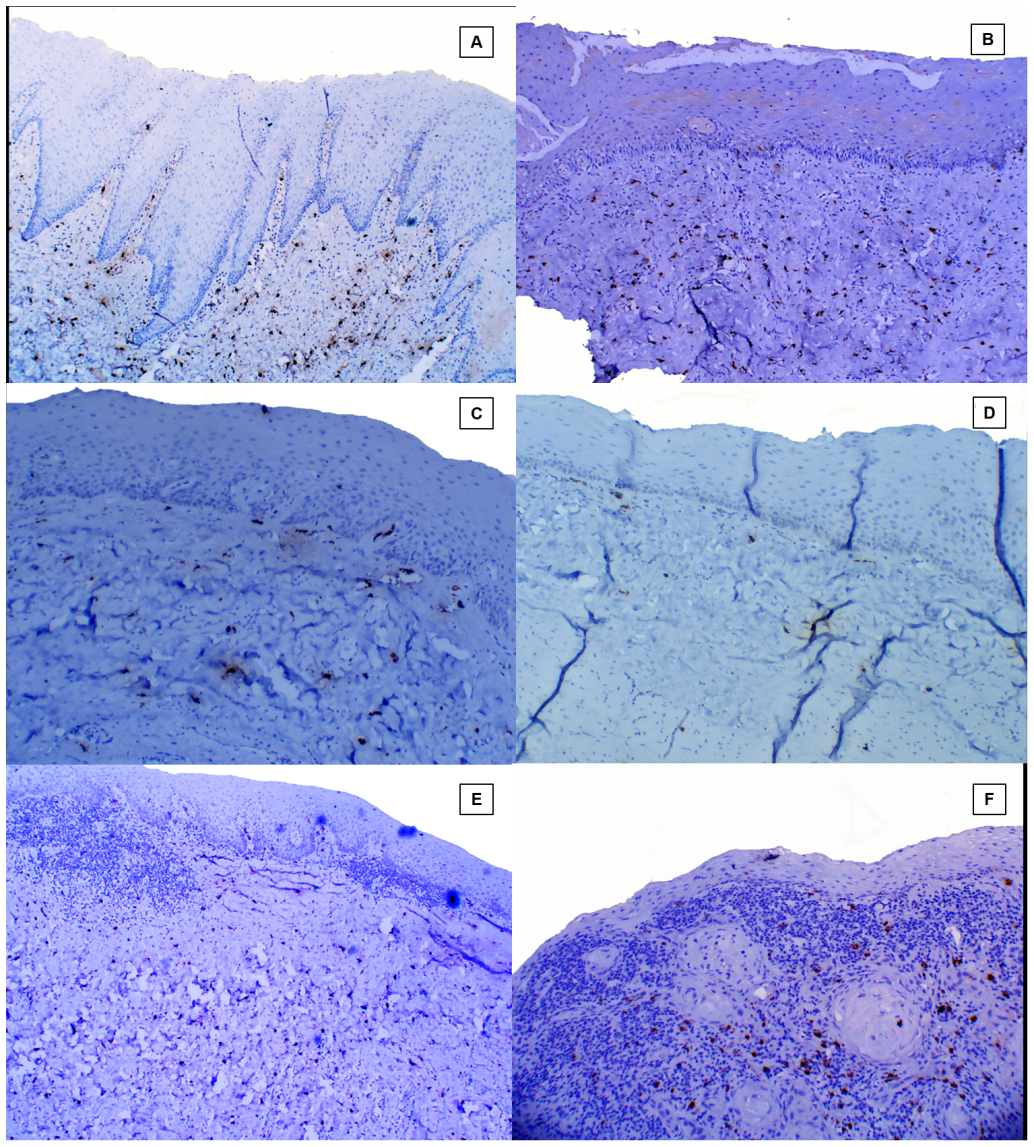
Subepithelial distribution of tryptase positive mast cells. **A**, Healthy control;. **B**, Stage 1 OSF. **C**, Stage 2 OSF. **D**, Stage 3 OSF. **E**, Stage 4 OSF. **F**, OSCC.

**Figure 6:**
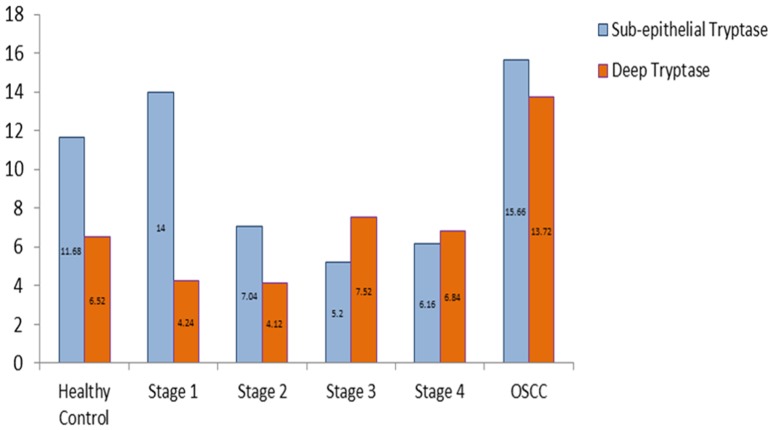
Mean value comparison of subepithelial and deep tryptase positive mast cells in healthy controls, different stages of OSF, and OSCC.

Stage I OSF showed a statistically significant increase in the number of tryptase positive MCs subepithelially as compared to other stages of OSF, however, no significant difference was noted between stage 2, 3 and 4 OSF (Figure. 6).

### Deep distribution of tryptase positive MCs in healthy controls, different stages of OSF and OSCC group (Figure. 7)

**Figure 7:**
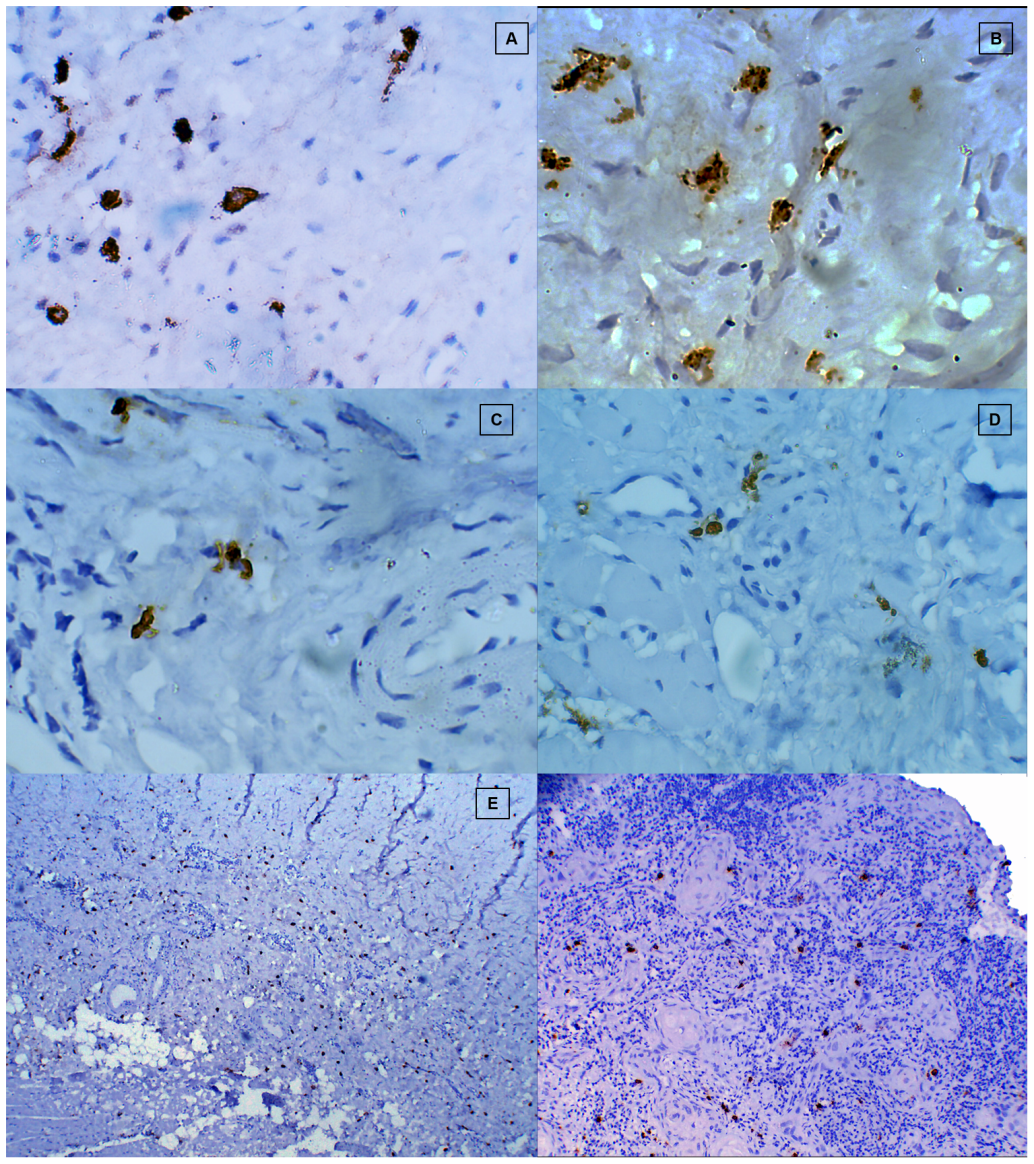
Deep distribution of tryptase positive mast cells. **A**, Healthy control. **B**, Stage 1 OSF. **C**, Stage 2 OSF. **D**, Stage 3 OSF. **E**, Stage 4 OSF. **F**, OSCC.

Deeper distribution of tryptase positive MCs was greater in healthy controls, OSCC group and advanced stages of OSF (Stage 3 and Stage 4) as compared to early stages of OSF (Stage 1 and stage 2) (Figure. 6).

### Subepithelial distribution of chymase positive MCs in healthy controls, different stages of OSF and OSCC group (Figure. 8)

**Figure 8:**
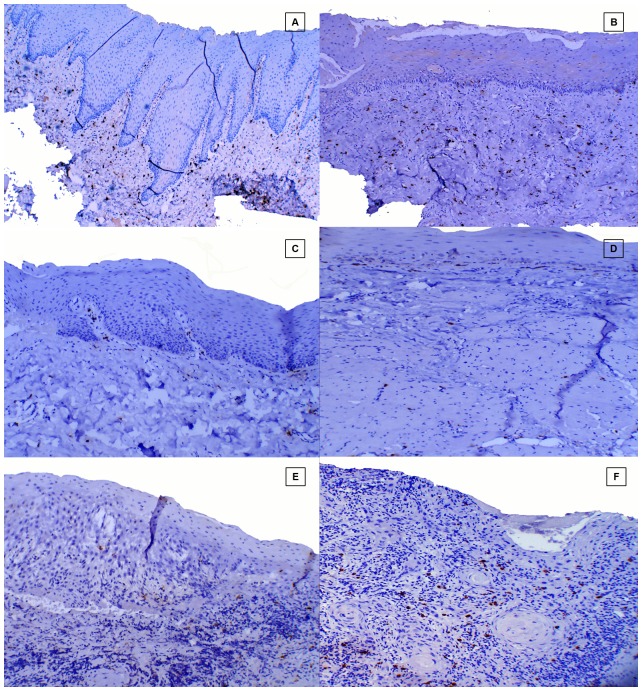
Subepithelial distribution of chymase positive mast cells. **A**, Healthy control. **B**, Stage 1 OSF. **C**, Stage 2 OSF. **D**, Stage 3 OSF. **E**, Stage 4 OSF. **F**, OSCC.

**Figure 9:**
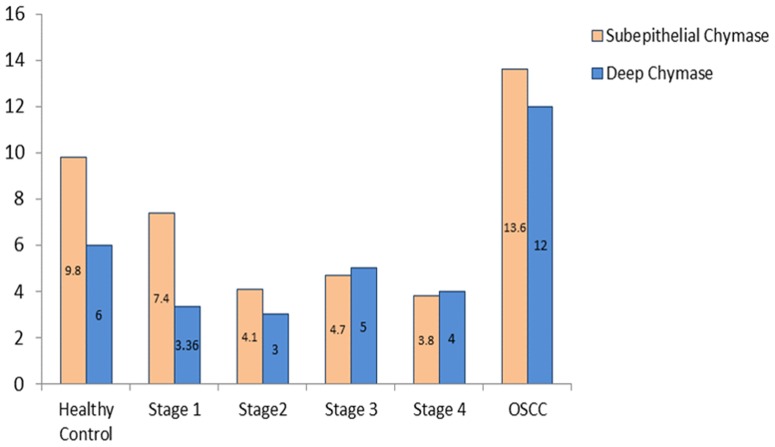
Mean value comparison of subepithelial and deep tryptase positive mast cells in healthy controls, different stages of OSF, and OSCC.

No statistically significant difference for the subepithelial distribution of chymase positive MCs was observed among different stages of OSF. A statistically significant increase in the number of subepithelial distribution of chymase positive MCs was noted in the OSCC group when compared to all the stages of OSF. Conversely, barring Stage 1 OSF, all stages of OSF showed a statistically decrease in the subepithelial distribution of chymase positive MCs when compared to healthy controls (Figure. 9).

### Deep distribution of chymase positive MCs in healthy controls, different stages of OSF and OSCC group (Figure. 10)

**Figure 10:**
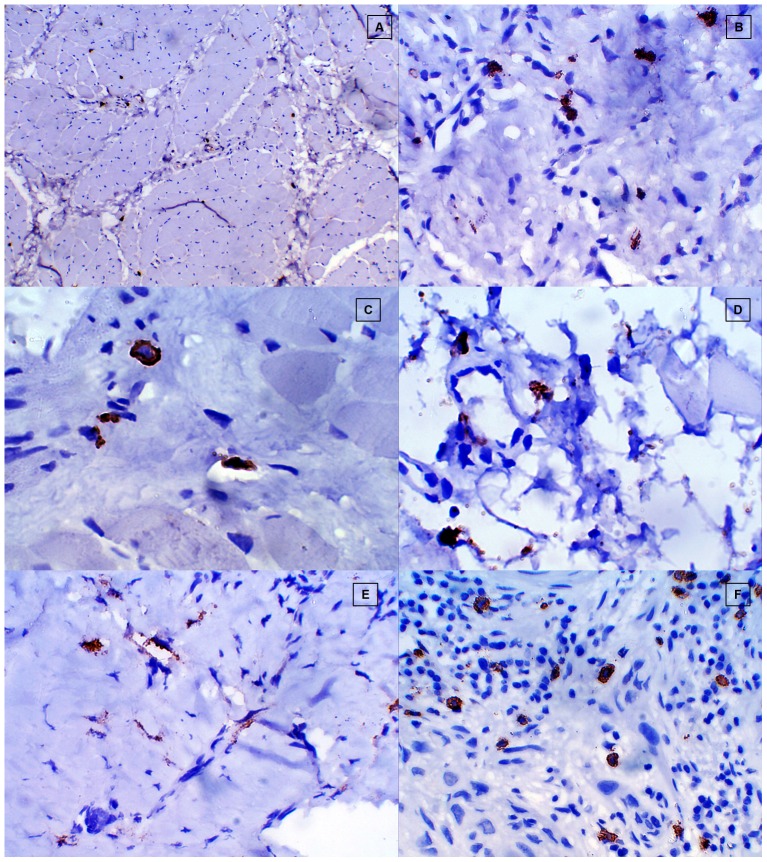
Deep distribution of distribution of chymase positive mast cells. A, Healthy control. B, Stage 1 OSF. C, Stage 2 OSF. D, Stage 3 OSF. E, Stage 4 OSF. F, OSCC.

A statistically significant decrease in the deeper distribution of chymase positive MCs was observed in all the stages of OSF when compared to healthy controls & OSCC group. OSCC group showed a statistically significant increase in chymase positive MCs in deeper areas when compared to healthy controls. However no statistically significant correlation was found for chymase positive MCs in deeper areas among different stages of OSF. OSCC group showed maximum number of chymase positive MCs in the deeper region when compared to all the other study groups (Figure. 9).

## Discussion

OSF is a chronic, inflammatory, premalignant fibrotic condition characterized by excessive deposition of collagen in the submucosa, leading to restricted mouth opening [19]. MC activation is a characteristic feature of chronic inflammation that may lead to fibrosis as a result of increased collagen synthesis by fibroblasts [37]. To the best of our knowledge this is the first study to emphasize the possible role of MC tryptase and chymase in OSF and its malignant transformation.

The enzyme profile of MCs in oral tissues resembles that of skin, with most MCs expressing the serine proteases, tryptase and chymase [38], [39]. Total MC distribution was higher in subepithelial region than the deeper connective tissue in all the study groups which was in accordance with other findings in literature [40]. From our results, it can be comprehended that OSF group showed the least while OSCC group showed the maximum number of tryptase positive MCs and chymase positive MCs irrespective of the area of distribution amongst the study groups. Thus, our findings support the idea of the possible role of MC tryptase and chymase in the pathogenesis of OSF and their role in upregulation of tumor angiogenesis during its malignant transformation.

### Role of MC tryptase and chymase in OSF

The fibrogenic cytokines influencing the fibrotic process are shown to play an important role in regulating fibroblast function, such as proliferation, migration, matrix synthesis and is likely to play a key role in regulating the initiation and progression of scarring in any fibrotic disease. The present study demonstrated a significantly higher subepithelial distribution of tryptase positive MCs than chymase positive MCs in the early stages of OSF against the increased distribution of tryptase positive MCs than chymase positive MCs in deeper zones of the advanced stages of OSF, which was in accordance with other findings in the literature [13], [14], [15], [16], [17], [18], [19]. This trend of initial increase in number of MC in stage I OSF followed by subsequent decrease in number of MC in later stages of OSF could be attributed to the initial inflammatory response of oral mucosa to the exogenous irritants and carcinogens like areca nut and tobacco. Subsequently, healing response in the form of fibrosis may be responsible for the decrease in total MC in Stage 2, 3 and 4 OSF.

MC tryptase is a major protease & produces mitogenic effects on various types of cultured cells such as smooth muscles and bronchial epithelial cells. Tryptase has in turn been described to activate TGF-β and collagenase, induce C3a and collagen mRNA, cleave type IV collagen, fibronectin, elastase, and proteoglycans, and induce fibroblast proliferation [41]. Tryptase has also been described to induce fibroblast procollagen mRNA upregulation. Studies have revealed that tryptase is a strong mitogen in its own right, but it synergizes its action with more traditional growth factors such as PDGF & FGF. Other investigators have shown that tryptase stimulates fibroblast chemotaxis and production of collagen [42], [43], however, the mechanism of the signaling event that mediates the fibrogenic effects of tryptase remains unclear.

A statistically significant decrease in subepithelial & deep distribution of chymase positive mast cells in all the stages of OSF (except in stage 1 OSF subepithelial distribution) in comparison to healthy controls and OSCC group made us to propose the possible role of MC chymase in the pathogenesis of OSF and its malignant transformation. Chymase can activate collagenase and stromelysin, destruct vitronectin and fibronectin, and induce fibroblast proliferation, suggesting an important role of this mast-cell-specific protease in tissue matrix turnover and renewal. Chymase has been shown to degrade the extra-cellular matrix (ECM) and basement membrane components, digest specific neuropeptides, and convert pro-IL-1B to its active molecule. Furthermore, it can cleave soluble stem cell factor (SCF) from its membrane form [44] and may thus contribute to the influx of MC precursors and to their in situ differentiation. Recent in-vitro studies have shown that MCs chymase also plays an important role in fibrosis [26]. Kofford et al [45] have reported that human chymase cleaves type I precollagen to form collagen fibrils in vitro. In addition, it has been reported that angiotensin II stimulates fibroblast proliferation through the activation of TGF–β1 [46]. Normally, several protease inhibitors within the connective tissue ensure tissue homeostasis by inhibiting excessive activities of chymase in the immediate MC environment. Such natural inhibitors are not known for MC-specific tryptase which is less active than chymase in connective tissue remodelling and fibroblast proliferation, resulting in shorter active life of chymase after secretion, thereby MC-derived tryptase can be considered as the main mediator stimulating fibroblast proliferation in the event of fibrosis [37].

**Figure 11:**
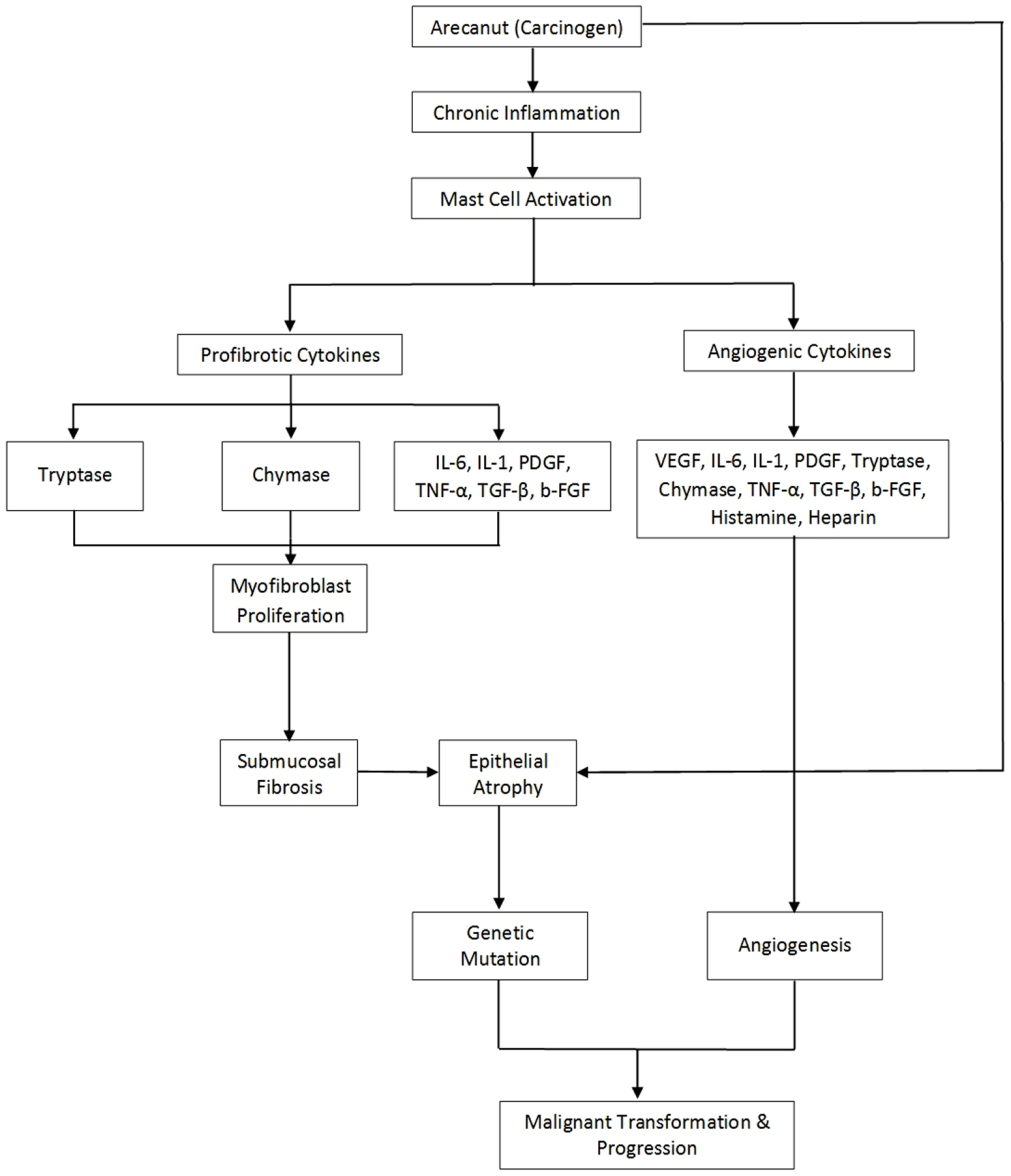
Schematic presentation of speculative hypothesis proposing possible role of mast cell tryptase and chymase in the pathogenesis of OSF and their possible role in tumor progression and malignant transformation of the overlying epithelium. IL-1, Interleukin-1. IL-6, Interleukin 6. TNF-α, Tumor necrosis factor α. TGF-β, Transforming growth factor β. PDGF, Platelet derived growth factor. VEGF, Vascular endothelial growth factor. B-FGF, Basic fibroblast growth factor.

MCs are a major source of other pro-fibrotic cytokines like bFGF [15], TGF-β [20], [22], [23], IL-6 [47], and their upregulation has been studied extensively in OSF. Thus, we suggest that a combination of tryptase and chymase along with the various fibrogenic cytokines seems to be an important factor in the development of OSF (Figure. 11).

From the above results it can be inferred that there is an altered balance in the subepithelial and deep distribution of tryptase positive MCs and chymase positive MCs as it progresses from healthy controls to OSF, thereby playing an important role in the pathogenesis of OSF. From the present study it can also be deduced that fibrosis in OSF may be initiating in the subepithelial zone in early stages of OSF (Stage 1 and stage 2) and gradually progressing towards the deeper muscle layer in advanced stages of OSF (Stage 3 and Stage 4). We observed a greater number of MCs in the muscle bundles of advanced stages than early stages of OSF. Based on our findings we assume that process of fibrosis in OSF remains uninterrupted since activated resident MCs continue to secrete fibrogenic & angiogenic cytokines even after the cessation of the areca nut chewing habit, resulting in irrevocable fibrosis.

### Role of MC tryptase and chymase in tumour progression

The pathogenesis of OSF and circumstances leading to its proven pre-cancerous outcome have always aroused curiosity but remained enigmatic till date. Fibroblast density and activation are increased in physiological responses, such as wound healing, and in disease states, such as fibrotic disorders and carcinogenesis [48], [49], [50], [51]. During epithelial carcinogenesis, fibroblasts have paracrine and autocrine interactions with resident and immune cells that include keratinocytes and MCs [52], [53], [54]. Fibroblasts can stimulate keratinocyte migration, proliferation, and malignant transformation [55], [56]. MCs and transformed epithelial cells stimulate fibroblasts to acquire an activated phenotype that favors tumor progression [56], [57], [58], [59]. Increased fibroblast density has been described previously during the formation of a reactive stroma in several neoplasia, including intraoral cancer and skin [49], [51], [57]. In the light of finding of the present study MC-derived tryptase can be considered as an important mediator of fibroblast activation and proliferation during malignant transformation of OSF since we observed a statistically significant increase in tryptase positive MCs in OSCC group as compared to other study groups [60].

Tumours require a blood supply for their expansive growth. Solid tumours, in order to outgrow the size of 2 mm^3^, demand for oxygen supply, a fact that makes necessary the formation of new microvasculature [61]. This progressive angiogenesis is the outcome of an imbalance between positive and negative angiogenic factors produced by both tumor and host cells. Among the host cells, which produce and release in a considerable quantity pro-angiogenic and angiogenic factors are MCs. The cancer stimulating mechanisms operated by MCs include participation in immunosuppression, the release of proangiogenic and mitogenic factors and involvement in the degradation of the extracellular matrix. MCs contain many angiogenic factors and a variety of cytokines, such as histamine, heparin, tryptase, chymase, PDGF, TNF-α, bFGF, TGF-β, IL-6 and vascular endothelial growth factor (VEGF) [21].Upregulation of the above pro-angiogenic cytokines in OSF has already been demonstrated in the literature [22], [23], [24], [25], [62]. The present study demonstrated a statistically significant increase in subepithelial distribution of chymase positive MCs in OSCC group when compared to healthy controls and OSF group. Chymase is known for its ability to promote extracellular matrix (ECM) degradation and for indirectly stimulating angiogenesis. Chymase activates latent matrix metalloproteinases (MMPs), including gelatinase B and procollagenase, which degrade components of epithelial basement membranes and ECM, respectively. These responses are essential for tumor invasion and metastasis [63]. We have already established the possible role of angiogenesis in malignant transformation of OSF by demonstrating CD34 positive blood vessels [63] and VEGF in atrophic epithelium of OSF [62].

As we have observed a significant increase in the tryptase and chymase positive MCs in OSCC group irrespective of the areas of distribution when compared to OSF group, it suggests that upregulation of MCs may play a crucial role in tumour progression during malignant transformation of atrophic epithelium in OSF (Figure.7.). A similar increase in the number of MC tryptase, a potent proangiogenic factor has been documented in various malignancies including oral cancers [26], [33], [64]. Hence, we would like to state that the event of fibrosis in the early OSF is probably a protective mechanism manifested in the form of excessive collagen deposition, primarily initiated in the connective tissue of the oral mucosa in order to prevent the deeper penetration of carcinogenic substances, while malignant transformation in the advanced OSF is entirely a different issue principally affecting the atrophic epithelium of OSF if the carcinogenic insult persists.

As part of the process of oral mucosal carcinogenesis in OSF, the connective tissue adjacent to the epithelium gets affected by the topically applied chemical carcinogen and plays a directive role, not yet understood, in the epithelial changes observed. It appears likely that extracellular matrix molecules, growth factors, angiogenic cytokines and proteinases co-operate in influencing epithelium via an autocrine proliferative effect on the atrophic epithelium, while paracrine stimulation of the vascular network may maintain survival and growth [19].

In summary, we present data indicating that MC tryptase and chymase contribute to the development of OSF and malignant transformation of the overlying epithelium. Several therapeutic approaches such as the use of MC stabilizer, blockade of stem cell factor or inhibitors of tryptase, chymase and TGF-β have already demonstrated some clinical utility in tissue fibrosis or inflammatory diseases, by inhibiting MC activation. Considering the important role of MC tryptase and chymase in the pathogenesis of OSF, further studies using either administration of a neutralizing antibody against tryptase and chymase or application of a MC tryptase knock-out mouse strategy in experimental models of OSF are required for more definitive determination of the role of tryptase and chymase in the development of OSF.

## References

[1] Kumar KK, Saraswathi TR, Ranganathan K, Devi UM, Joshua E (2007) Oral Submucous fibrosis; A clinicopathological study in Chennai. Ind J Dent Res 18: 106–111. 10.4103/0970-9290.33785 17687172

[2] Rajalalitha P, Vali S (2005) Molecular pathogenesis of oral submucous fibrosis – a Collagen metabolic disorder. J Oral Pathol Med 34: 321–328. 15946178 10.1111/j.1600-0714.2005.00325.x

[3] Angadi PV, Kale AD, Hallikerimath S (2011) Evaluation of myofibroblasts in oral submucous fibrosis: correlation with disease severity. J Oral Pathol Med 40: 208–213. 21198872 10.1111/j.1600-0714.2010.00995.x

[4] Punnya V, Angadi SS (2011) Role Areca nut in pathogenesis of oral submucous fibrosis: revisited. Oral and Maxillofac Surg 15: 1–9. 20376683 10.1007/s10006-010-0219-8

[5] Cairns JA, Walls AF (1997) Mast cell tryptase stimulates the synthesis of type I collagen in human lung fibroblasts. J Clin Invset 99: 1313–1321. 10.1172/JCI119290 PMC507947 9077541

[6] Choi KL, Claman HN (1987) Mast cells, fibroblasts and fibrosis. New clues to the riddle of mast cells. Immunol Res 6: 145–152. 3316438 10.1007/BF02918088

[7] Akers IA, Parsons M, Hill MR, Hollenberg MD, Sanjar S (2000) Mast cell tryptase stimulates human lung fibroblast proliferation via protease-activated receptor-2. Am J Physiol Lung Cell Mol Physiol 278: L193–L201. 10645907 10.1152/ajplung.2000.278.1.L193

[8] Frungeiri MB, Weidinger S, Meineke V, Kohn FM, Mayerhofer A (2002) Proliferative action of mast-cell tryptase is mediated by PAR2, COX2, prostaglandins, and PPAR: Possible relevance to human fibrotic disorders. Proc Natl Acad Sci USA 99: 15072–15077. 12397176 10.1073/pnas.232422999 PMC137545

[9] Farrell DJ, Hines JE, Walls AF, Kelly PJ, Bennett MK, et al (1995) Intrahepatic mast cells in chronic liver diseases. Hepatology 22: 1175–1181. 7557869 10.1016/0270-9139(95)90627-4

[10] Akimoto S, Ishikawa O, Igarashi Y, Kurosawa M, Miyachi Y (1998) Dermal mast cells in scleroderma: their skin density, tryptase/chymase phenotypes and degranulation. Br J Dermatol 138: 399–406. 9580789 10.1046/j.1365-2133.1998.02114.x

[11] Wang HW, Tedla N, Hunt JE, Wakefield D, McNeil HP (2005) Mast cell accumulation and cytokine expression in the tight skin mouse model of scleroderma. Exp Dermatol 14: 295–302. 15810888 10.1111/j.0906-6705.2005.00315.x

[12] Roberts IS, Brenchley PE (2000) Mast cells: the forgotten cells of renal fibrosis. J Clin Pathol 53: 858–862. 11127270 10.1136/jcp.53.11.858 PMC1731105

[13] Bhatt AP, Dholakia HM (1997) Mast cell density in oral submucous fibrosis. J Ind Dent Assoc 49: 187–191.

[14] Ankle MR, Kale AD, Nayak R (2007) Mast cells are increased in leukoplakia, oral submucous fibrosis, oral lichen planus and oral squamous cell carcinoma. J Oral and Maxillofac Pathol 11: 18–22.

[15] Bishen KA, Radhakrishnan R, Satyamoorthy K (2008) The role of basic fibroblast growth factor in oral submucous fibrosis pathogenesis. J Oral Pathol Med 37: 402–411. 18298475 10.1111/j.1600-0714.2008.00649.x

[16] Sabarinath B, Sriram G, Saraswathi TR, Sivapathasundharam B (2011) Immunohistochemical evaluation of mast cells and vascular endothelial proliferation in oral submucous fibrosis. Ind J Dent Res 22: 116–121. 10.4103/0970-9290.80009 21525689

[17] Pujari R, Vidya N (2013) Mast cell density in oral submucous fibrosis: a possible role in pathogenesis. Int J Health Sci, Qassim University 7: 23–29. 10.12816/0006017 PMC3612412 23559902

[18] Chavan S, Deshmukh SR (2013) Quantitative analysis of mast cells in oral submucous fibrosis. Al Ameen J Med Sci 6: 144–149.

[19] Khatri MJ, Desai RS, Mamatha GS, Kulkarni M, Khatri J (2013) Immunohistochemical expression of mast cells using c- kit in various grades of oral submucous fibrosis. ISRN Pathology Article ID 543976. 5 Pages.

[20] Kale AD, Mane DR, Shukla D (2013) Expression of transforming growth factor β and its correlation with lipodystrophy in oral submucous fibrosis: an Immunohistochemical study. Med Oral Pathol Oral Cir Bucal 18: e12–18. 10.4317/medoral.18226 PMC3548630 22926483

[21] Metcalfe DD, Baram D, Mekori Y (1997) Mast cells. Physiol Rev 77: 1033–1079. 9354811 10.1152/physrev.1997.77.4.1033

[22] Haque MF, Harris M, Meghji S, Speight PM (1997) An immunohistochemical study of oral submucous fibrosis. J Oral Pathol Med 26: 75–82. 9049906 10.1111/j.1600-0714.1997.tb00025.x

[23] Haque MF, Harris M, Meghji S, Barrett AW (1998) Immunohistochemical localization of cytokines and growth factors in oral submucous fibrosis. Cytokine 10: 713–719. 9770333 10.1006/cyto.1997.0342

[24] Tsai CC, Chen CC, Lin CC, Chen CH, Lin TS, et al (1999) Interleukin-1 beta in oral submucous fibrosis, verrucous hyperplasia and squamous cell carcinoma tissues. Kaohsiung J Med Sci 15: 513–519. 10561975

[25] Khan I, Agarawal P, Thangiam GS, Radhesh R, Rao SG, et al (2011) Role of TGF-β and BMP 7 in pathogenesis of oral submucous fibrosis. Growth Factor 29: 119–127. 10.3109/08977194.2011.582839 21591998

[26] Fukushima H, Ohsawa M, Ikura Y, Naruka T, Sugama Y, et al (2006) Mast cells in diffuse large B-cell lymphoma: their role in fibrosis. Histopathol 49: 498–505. 10.1111/j.1365-2559.2006.02534.x 17064296

[27] Folkman J (1985) Tumour angiogenesis. Adv Cancer Res 43: 203. 10.1016/s0065-230x(08)60946-x 2581424

[28] Folkman J, Shing Y (1992) Angiogenesis. J Biol Chem 267: 10931–10934. 1375931

[29] Yoshiji H, Gomez DE, Shibuya M, Thorgeirsson UP (1996) Expression of vascular endothelial growth factor, its receptor, and other angiogenic factors in human breast cancer. Cancer Res 56: 2013–2016. 8616842

[30] Weidner N, Semple JP, Welch WR, Folkman J (1991) Tumor angiogenesis and metastasis—correlation in invasive breast carcinoma. N Engl J Med 324: 1–8. 10.1056/NEJM199101033240101 1701519

[31] Tanigawa N, Amaya H, Matsumura M, Shimomatsuya T (1997) Correlation between expression of vascular endothelial growth factor and tumor vascularity, and patient outcome in human gastric carcinoma. J Clin Oncol 15: 826–832. 9053510 10.1200/JCO.1997.15.2.826

[32] Kyzas PA, Stefanou D, Batistatou A, Agnantis NJ (2005) Prognostic significance of VEGF immunohistochemical expression and tumor angiogenesis in head and neck squamous cell carcinoma. J Cancer Res Clin Oncol 131: 624–630. 16044346 10.1007/s00432-005-0003-6 PMC12161278

[33] Iamaroon A, Pongsiriwet S, Jittidecharaks S, Pattanaporn K, Prapayasatok S, et al (2003) Increase of mast cells and tumor angiogenesis in oral squamous cell carcinoma. J Oral Pathol Med 32: 195–199. 12653857 10.1034/j.1600-0714.2003.00128.x

[34] Elpek GO, Gelen T, Aksoy NH, Erdoğan A, Dertsiz L, et al (2001) The prognostic relevance of angiogenesis and mast cells in squamous cell carcinoma of the oesophagus. J Clin Pathol 54: 940–944. 11729214 10.1136/jcp.54.12.940 PMC1731336

[35] Irani AA, Schechter NM, Craig SS, DeBlois G, Schwartz LB (1986) Two types of human mast cells have distinct neutral protease compositions. Proc Natl Acad Sci USA 83: 4464–4468. 3520574 10.1073/pnas.83.12.4464 PMC323754

[36] Rojas IG, Spencer MI, Martinez A, Marurellia MA, Rudolph MI (2005) Characterization of mast Cell subpopulation in lip Cancer. J Oral Pathol Med 34: 268–273. 15817069 10.1111/j.1600-0714.2004.00297.x

[37] Caughey GH (2007) Mast cell tryptase and chymase in inflammation and host defense. Immunol Rev 217: 141–154. 17498057 10.1111/j.1600-065X.2007.00509.x PMC2275918

[38] Walsh LJ (2003) Mast cells and oral inflammation. Crit Rev in Oral Biol Med 14: 188–198. 12799322 10.1177/154411130301400304

[39] Walsh LJ, Davis MF, Xu LJ, Savage NW (1995) Relationship between mast cell degranulation and inflammation in the oral cavity. J Oral Pathol Med 24: 266–272. 7562663 10.1111/j.1600-0714.1995.tb01180.x

[40] Roukonen H, Hietanen J, Malmstrom M, Sane J, Hayrinen RI, et al (1993) Peripheral nerves and mast cells in normal buccal mucosa. J Oral Pathol Med 22: 30–34. 7678295 10.1111/j.1600-0714.1993.tb00116.x

[41] Algermissen B, Bauer F, Schadendorf, Kroopp JD, Czarnetzki BM (1994) Analysis of mast cell subpopulations (MCT, MCTC) in cutaneous inflammation using novel enzyme-histochemical staining techniques. Exp Dermatol 3: 290–297. 7538409 10.1111/j.1600-0625.1994.tb00291.x

[42] Gruber BL, Kew RR, Jelaska A, Marcheses MJ, Garlick J, et al (1997) Human mast cells activate fibroblasts: Tryptase is a fibrogenic factor stimulating collagen messenger ribonucleic acid synthesis and fibroblast chemotaxis. J Immunol 158: 2310–2317. 9036979

[43] Ruoss SJ, Thomas H, Caughey GH (1991) Mast cell tryptase is a mitogen for cultured fibroblasts. J Clin Invest 88: 439–499. 10.1172/JCI115330 PMC295370 1864960

[44] Longley BJ, Tyrrell L, Ma Y, Williams DA, Halaban R, et al (1997) Chymase cleavage of stem cell factor yields a bioactive, soluble product. Proc Natl Acad Sci USA 94: 9017–9021. 9256427 10.1073/pnas.94.17.9017 PMC23007

[45] Kofford MW, Schwartz LB, Schecter NW, Yager DR, Diegelmann RF, et al (1997) Cleavage of type II precollagen by human mast cell chymase initiates collagen fibril formation and generates a unique carboxyl-terminal propeptide. J Biol Chem 272: 7127–7131. 9054407 10.1074/jbc.272.11.7127

[46] Lingstedt KA, Wang Y, Shiota N, Saarinen J, Hytiasnen M, et al (2001) Activation of paracrine TGF-β1 signalling upon stimulation and degranulation of rat serosal mast cells: a novel function for chymase. FASEB J 15: 1377–1388. 11387235 10.1096/fj.00-0273com

[47] Chen CC, Huang JF, Tsai CC (1995) In vitro production of interleukin-6 by human gingival, normal buccal mucosa, and oral submucous fibrosis fibroblasts treated with betel-nut alkaloids. Kaohsiung J Med Sci 11: 604–614. 7490793

[48] Martin P (1997) Wound healing-aiming for perfect skin regeneration. Science 276: 75–81. 9082989 10.1126/science.276.5309.75

[49] Kalluri R, Zeisberg M (2006) Fibroblasts in cancer. Nat Rev Cancer 6: 392–401. 16572188 10.1038/nrc1877

[50] Wynn TA (2008) Cellular and molecular mechanisms of fibrosis. J Pathol 214: 199–210. 18161745 10.1002/path.2277 PMC2693329

[51] Thode C, Jorgensen TG, Dabelsteen E, Mackenzie I, Dabelsteen S (2011) Significance of myofibroblasts in oral squamous cell carcinoma. J Oral Pathol Med 40: 201–207. 21342271 10.1111/j.1600-0714.2010.00999.x

[52] Sorrell JM, Caplan AI (2004) Fibroblast heterogeneity: more than skin deep. J Cell Sci 117(: 667–675. 14754903 10.1242/jcs.01005

[53] Taun TL, Keller LC, Sun D, Nimni ME, Cheung D (1994) Dermal fibroblasts activate keratinocyte outgrowth on collagen gels. J Cell Sci 107: 2285–2289. 7983187 10.1242/jcs.107.8.2285

[54] Trautmann A, Krohne G, Brocker EB, Klein CE (1998) Human Mast cells augment fibroblast proliferation by heterotypic cell-cell adhesion and action of IL-4. J Immunol 160: 5053–5057. 9590255

[55] Krtolica A, Parrinello S, Lockett S, Desprez PY, Campisi J (2001) Senescent fibroblasts promote epithelial cell growth and tumorigenesis: a link between cancer and aging. Proc Natl Acad Sci USA 98: 12072–12077. 11593017 10.1073/pnas.211053698 PMC59769

[56] Bhowmick NA, Neilson EG, Moses HL (2004) Stromal fibroblasts in cancer initiation and progression. Nature 432: 332–337. 15549095 10.1038/nature03096 PMC3050735

[57] Coussens LM, Raymond WW, Bergers G (1999) Inflammatory mast cells upregulate angiogenesis during squamous epithelial carcinogenesis. Genes Dev 13: 1382–1397. 10364156 10.1101/gad.13.11.1382 PMC316772

[58] Artuc M, Steckelings UM, Henz BM (2002) Mast cell-fibroblast interactions: human mast cells as source and inducers of fibroblast and epithelial growth factors. J Invest Dermatol 118: 391–395. 11874475 10.1046/j.0022-202x.2001.01705.x

[59] Mueller MM, Fusenig NE (2004) Friends or foes–bipolar effects of the tumour stroma in cancer. Nat Rev Cancer 4: 839–849. 15516957 10.1038/nrc1477

[60] Rojas IG, Boza YV, Spencer ML, Flores M, Martinez A (2012) Increased fibroblast density in actinic cheilitis: association with tryptase-positive mast cells, actinic elastosis and epithelial p53 and COX-2 expression. J Oral Pathol Med 41: 27–33. 21689160 10.1111/j.1600-0714.2011.01057.x

[61] Folkman J (2000) Tumor Angiogenesis.In: Cancer Medicine, 5th ed, London: Decker pp 13–52.

[62] Desai RS, Mamatha GS, Khatri MJ, Shetty SJ (2012) Immunohistochemical expression of vascular endothelial growth factor (VEGF) and its possible role in tumor progression during malignant transformation of atrophic epithelium in oral submucous fibrosis. Current Angiogenesis 1: 347–53.

[63] Desai RS, Mamatha GS, Khatri MJ, Shetty SJ (2010) Immunohistochemical expression of CD34 for characterization and quantification of mucosal vasculature and its probable role in malignant transformation of atrophic epithelium in oral submucous fibrosis. Oral Oncol 46: 553–558. 20538504 10.1016/j.oraloncology.2010.04.004

[64] Benitez-Bribiesca L, Wong A, Utera D, Castellanos E (2001) The role of mast cell tryptase in neoangiogenesis of premalignant and malignant lesion of uterine cervix. J Histochem and Cytochem 49: 1061–1062. 11457936 10.1177/002215540104900816

